# Quantitative assessment of the associations between DNA repair gene XRCC3 Thr241Met polymorphism and pancreatic cancer

**DOI:** 10.1186/s12957-024-03450-1

**Published:** 2024-06-25

**Authors:** Wenjing Wu, Sen Xu, Lingzhi Chen, Chaomin Ji, Tianyu Liang, Mangmang He

**Affiliations:** 1 General Surgery, Cancer Center, Department of Gastrointestinal and Pancreatic Surgery, Department of nursing, Zhejiang Provincial People’s Hospital(Affiliated People’s Hospital), Hangzhou Medical College, Hangzhou, Zhejiang China; 2 Second Clinical Medical School, Zhejiang Chinese Medical University, Hangzhou, China; 3 Emergency and Critical Care Center, Intensive Care Unit, Zhejiang Provincial People’s Hospital (Affiliated People’s Hospital), Hangzhou Medical College, No.158 Shangtang Road, Hangzhou, Zhejiang China; 4 Department of the Operating Room, Zhejiang Provincial People’s Hospital (Affiliated People’s Hospital), Hangzhou Medical College, Hangzhou, Zhejiang China

**Keywords:** Pancreatic cancer, XRCC3, Gene polymorphism, Meta-analysis

## Abstract

**Background:**

Prior research exploring the correlation between the XRCC3 Thr241Met polymorphism and the susceptibility to pancreatic cancer has yielded conflicting outcomes. To date, there has been a notable absence of studies examining this polymorphism. The primary aim of the current investigation is to elucidate the potential role of the XRCC3 Thr241Met polymorphism as a risk factor in the development of pancreatic cancer.

**Methods:**

The comprehensive literature search was meticulously conducted across primary databases, including PubMed, Embase, and CNKI (China National Knowledge Infrastructure), spanning from the inception of each database through January 2024. To synthesize the data, a meta-analysis was performed using either a fixed or random-effects model, as appropriate, to calculate the odds ratios (ORs) and their corresponding 95% confidence intervals (CIs).

**Results:**

The analysis revealed significant associations between the XRCC3 Thr241Met polymorphism and an increased risk of pancreatic cancer. This was evidenced through various genetic model comparisons: allele contrast (T vs. C: OR = 0.77, 95% CI = 0.70–0.86, *P* < 0.001), homozygote comparison (TT vs. CC: OR = 0.71, 95% CI = 0.58–0.88, *P* = 0.001), heterozygote comparison (TC vs. CC: OR = 0.67, 95% CI = 0.52–0.87, *P* = 0.003), and a dominant genetic model (TT/TC vs. CC: OR = 0.68, 95% CI = 0.57–0.81, *P* < 0.001). Additionally, subgroup analyses based on ethnicity disclosed that these associations were particularly pronounced in the Caucasian population, with all genetic models showing significance (*P* < 0.05).

**Conclusions:**

The XRCC3 Thr241Met polymorphism has been identified as contributing to a reduced risk of pancreatic cancer in the Caucasian population. This finding underscores the need for further research to validate and expand upon our conclusions, emphasizing the urgency for continued investigations in this domain.

## Introduction

Pancreatic cancer ranks as one of the most lethal malignancies globally, currently holding the fourth position in cancer-related fatalities [1]. In 2012, there were approximately 178,000 new cases reported [2]. Forecasts suggest that by 2030, pancreatic cancer may become the second leading cause of death from malignant tumors [3, 4]. The incidence and mortality rates of pancreatic cancer exhibit considerable variation across different regions. A study encompassing 54 countries and regions from 1980 to 2007 revealed the highest mortality rates in Northern Europe and the Baltic Sea region, averaging 9.5 per 100,000, with Lithuania reporting the highest at 11.1 per 100,000. Conversely, lower mortality rates were observed in Hong Kong, Japan, Latin America, the United States, and Russia, with Venezuela having the lowest rate in Latin America at 2.9 per 100,000.

The insidious onset of pancreatic cancer, coupled with its nonspecific early symptoms, poses significant diagnostic challenges. It is often misdiagnosed as gastroduodenal ulcers, diabetes, or other conditions. Consequently, most patients are diagnosed at an advanced stage. The low rate of surgical resection, coupled with limited treatment options and negligible efficacy, results in a dismal five-year survival rate of less than 6% [5], posing a grave threat to public health. Understanding the factors related to the development of pancreatic cancer is therefore crucial for reducing its incidence and mortality.

The exact pathogenesis of pancreatic cancer remains elusive, and there is a lack of effective screening and early diagnosis techniques. Pancreatic cancer arises from a complex interplay of genetic and environmental factors. During the development of pancreatic cancer, mutations in repair genes alter their function and expression levels, triggering mutations in oncogenes and tumor suppressor genes that regulate the disease. This accumulation of changes eventually leads to pancreatic cancer.

XRCC3 is a DNA repair gene primarily involved in the repair of double-strand DNA breaks. Its deletion or mutation significantly increases susceptibility to DNA damage factors, contributing to the onset of malignant tumors like lung cancer. The C to T mutation at nucleoside acid 18,067 in exon of XRCC3 results in a codon change at position 241 from threonine (Thr) to methionine (Met), disrupting the protein’s normal conformation and potentially enhancing susceptibility to certain tumors. The XRCC3 Thr241Met gene polymorphism has been linked to pancreatic cancer development. However, due to variations in study populations, sample sizes, genetic backgrounds, and environmental exposures, the results of independent case-control studies have been inconsistent. Therefore, this study aims to apply evidence-based medicine principles and methods to conduct a comprehensive analysis of all published studies on the relationship between XRCC3 Thr241Met polymorphism and genetic susceptibility to pancreatic cancer.

## Materials and methods

### Data Collection

Using a variety of well-known databases, including PubMed, EMBASE, the Cochrane Library, Google Scholar, and CNKI (China National Knowledge Infrastructure), two separate writers carried out extensive searches. The search period ran from each database’s creation until January 2024. The terms “pancreatic cancer,” “polymorphism” or “polymorphisms,” and “XRCC3” were used to deliberately narrow the search.

### Inclusion and exclusion criteria

The following exacting standards were established for inclusion in this analysis: Studies that explicitly evaluate the relationship between the XRCC3 Thr241Met polymorphism and pancreatic cancer fall into two categories: (a) case-control studies; and (b) studies with enough information to compute odds ratios (ORs) and their 95% confidence intervals (CIs). On the other hand, the exclusion criteria were similarly strict and included: (a) studies that did not follow the case-control design; (b) studies that did not have enough data to calculate the OR and 95% CI; and (c) studies that used animals as experimental subjects.

### Data extraction and methodological quality assessment

Following strict adherence to the predetermined inclusion and exclusion criteria, the first and second authors carefully went through and assessed all available data and information. When the first and second writers couldn’t agree on anything, they discussed it and came to a mutually agreed-upon decision by consulting the corresponding author. Three primary components comprised the main assessment criteria: the evaluation of exposure outcomes and variables (0–3 points); comparability between groups (0–2 points); and the selection of cases and controls (0–4 points). References 6–18 provide transparent methods and procedures used in this review that are cross-referenceable with previously published material [6–18].

### Statistical analysis

With the aid of odds ratios (ORs) and 95% confidence intervals (CIs), the degree of correlation between the XRCC3 Thr241Met polymorphism and the risk of pancreatic cancer was statistically determined. Both the Q-statistic and I2 statistics were used to assess the level of heterogeneity among the studies. Four genetic models were used to investigate this relationship, in line with other research. The degree of observed heterogeneity dictated which model to use: a fixed-effects model or a random-effects model [19, 20]. Sensitivity analyses and publication bias evaluations were carried out according to the protocols set forth in earlier meta-analyses [13–18]. The software Stata 15.0 was used for all statistical analyses. According to the Preferred Reporting Items for Systematic Reviews and Meta-Analyses (PRISMA) 2009 standards, this meta-analysis was carried out meticulously and reported.

## Results

### General information

**Fig. 1:**
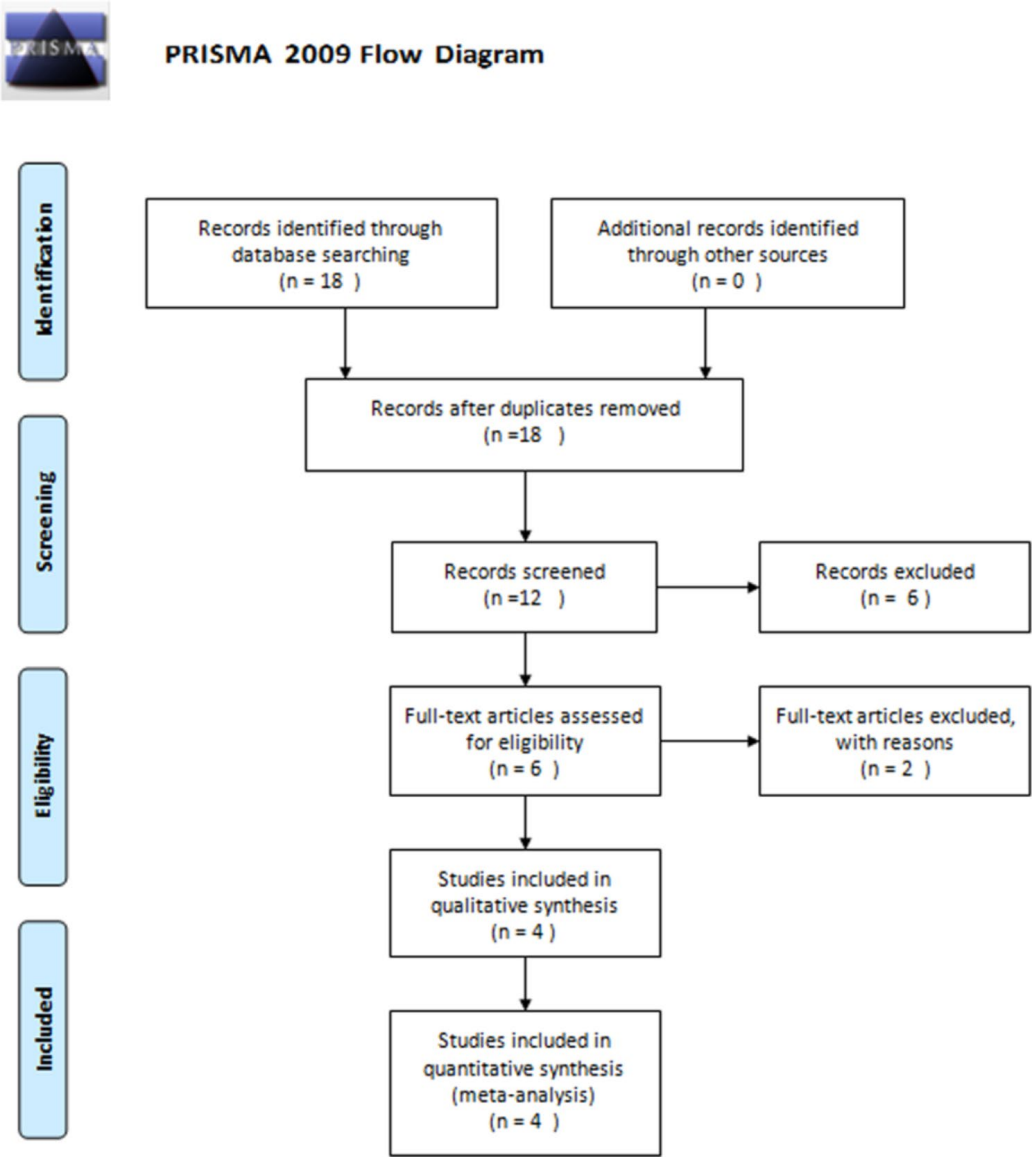
PRISMA 2009 Flow Diagram

The search methodology for this meta-analysis is outlined in the PRISMA 2009 Flow Diagram (Fig. 1). Four studies in all were eventually included. A thorough summary of the most important facts and data from these investigations is shown in Table 1. Geographically, two studies from the United States, one each from France and Poland made up the included literature. Numerous genotyping techniques, such as the Masscode methodology, allele-specific assays, and Taqman assays, were used in these investigations. These studies were published between the years of 2006 and 2016, and the control groups were hospital or population-based. Every control group’s genotype frequency was in line with Hardy-Weinberg Equilibrium (HWE). These studies’ sample sizes ranged from 33 to 1120 people. The Newcastle-Ottawa Scale (NOS) scores are shown in Table 2, with an average score of 7.9 indicating a high quality across all research.

**Table 1:** General information of eligible studies enrolled in the meta-analysis

Literature	Ethnics(Country)	Genotyping method	Control Origin	Sample capacity	Matching standard	HWE conformity	NOS
Donghui(2006a)	Caucasian(United states)	Masscode technique	HB	332/436	Age, sex, ethnicity	Yes	9
Donghui(2006b)	Hispanics(United states)	Masscode technique	HB	22/20	Age, sex, ethnicity	Yes	8
Donghui(2006c)	African(United states)	Masscode technique	HB	19/25	Age, sex, ethnicity	Yes	8
Jiao(2008a)	Caucasian (United states)	allele specific assay	HB	416/436	Age, sex, ethnicity	Yes	9
Jiao(2008b)	Hispanics (United states)	allele specific assay	HB	26/20	Age, sex, ethnicity	Yes	8
Jiao(2008c)	African (United states)	allele specific assay	HB	17/16	Age, sex, ethnicity	Yes	8
Duell(2008a)	Caucasian(France)	Taqman assay	PB	260/860	Age, sex, ethnicity	Yes	8
Duell(2008b)	African (France)	Taqman assay	PB	48/104	Age, sex, ethnicity	Yes	7
Renata(2016)	Caucasian(Poland)	Taqman assay	HB	101/103	Age, sex, ethnicity	Yes	8

PB: Population-based; HB: Hospital-based; HWE: Hardy-Weinberg equilibrium; NOS: Newcastle-Ottawa Scale

**Fig. 2:**
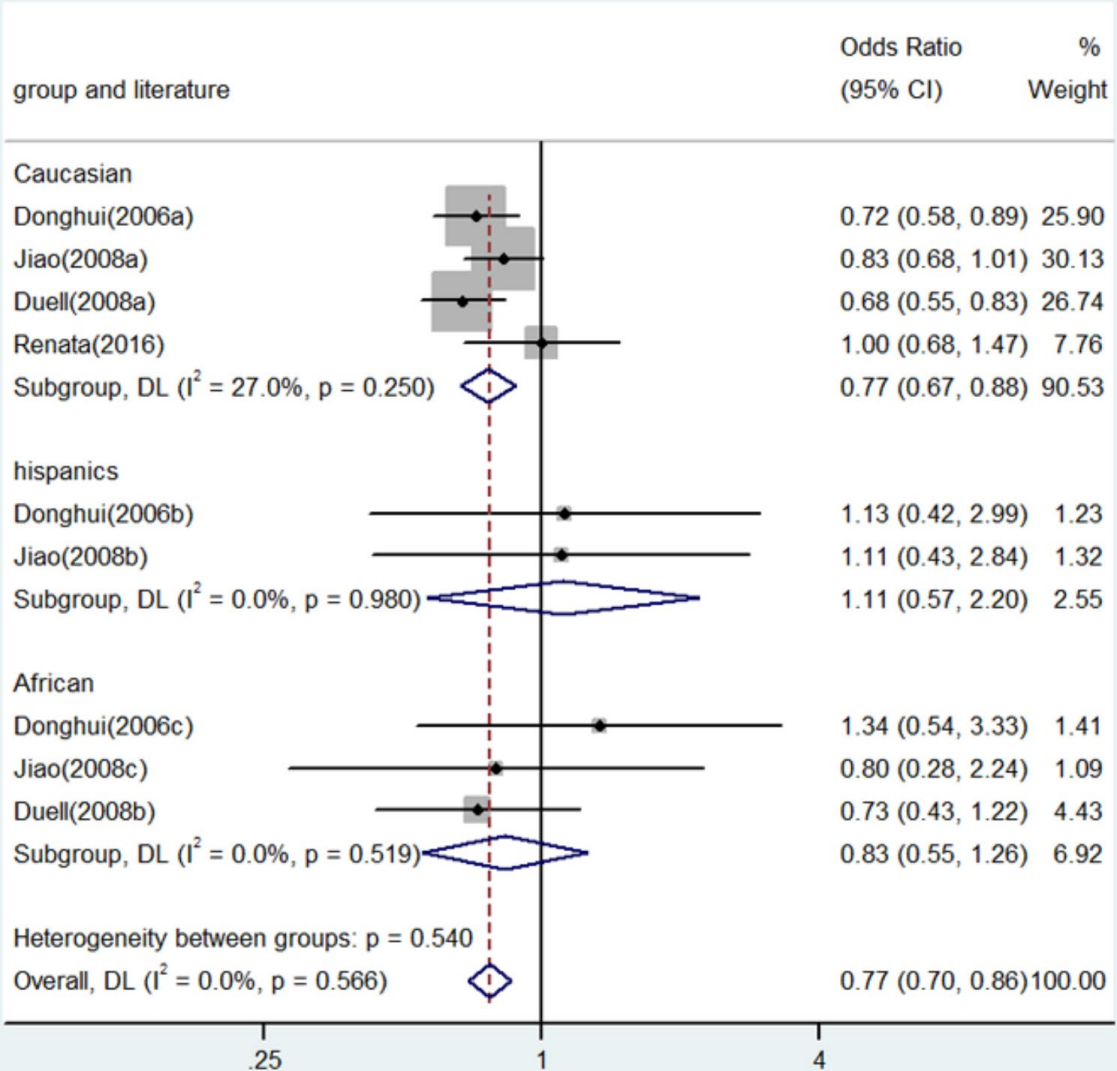
Forest plot for the associations between XRCC3 Thr241Met polymorphism and pancreatic cancer risk through homozygote comparison (TT vs. CC). OR, odds ratio; CI, confidence interval

**Table 2:** Quality assessment of the seven case–control studies according to the Newcastle-Ottawa Scale

Literature	selection of enrolled study subjects	between-group comparability	exposure outcomes and factors	Total
Donghui(2006a)	3	3	3	9
Donghui(2006b)	3	2	2	8
Donghui(2006c)	3	3	2	8
Jiao(2008a)	3	3	3	9
Jiao(2008b)	3	2	3	8
Jiao(2008c)	3	3	2	8
Duell(2008a)	3	3	2	8
Duell(2008b)	2	3	2	7
Renata(2016)	3	3	2	8
Average	2.9	2.7	2.3	7.9

**Fig. 3:**
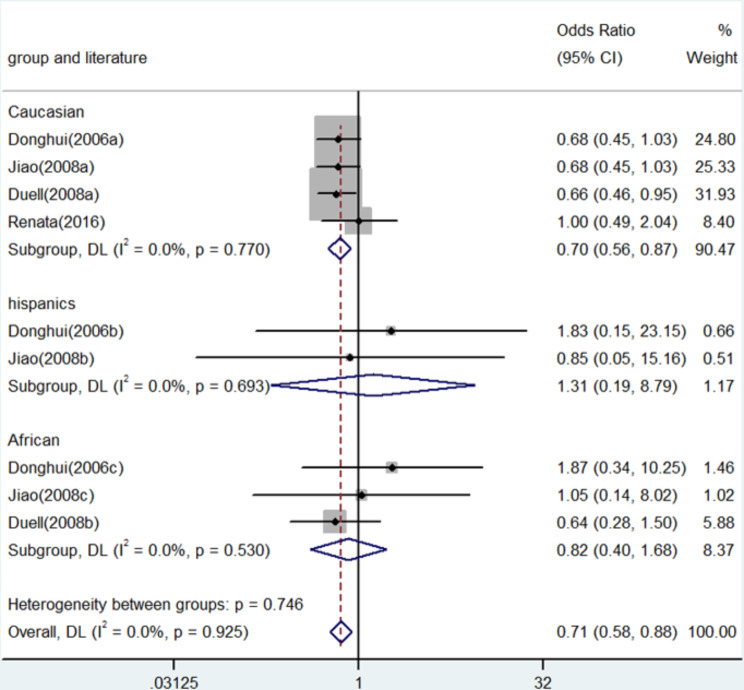
Forest plot for the associations between XRCC3 Thr241Met polymorphism and pancreatic cancer risk through allele contrast (T vs. C). OR, odds ratio; CI, confidence interval

### Allele and genotype-wide meta-analysis

**Fig. 4:**
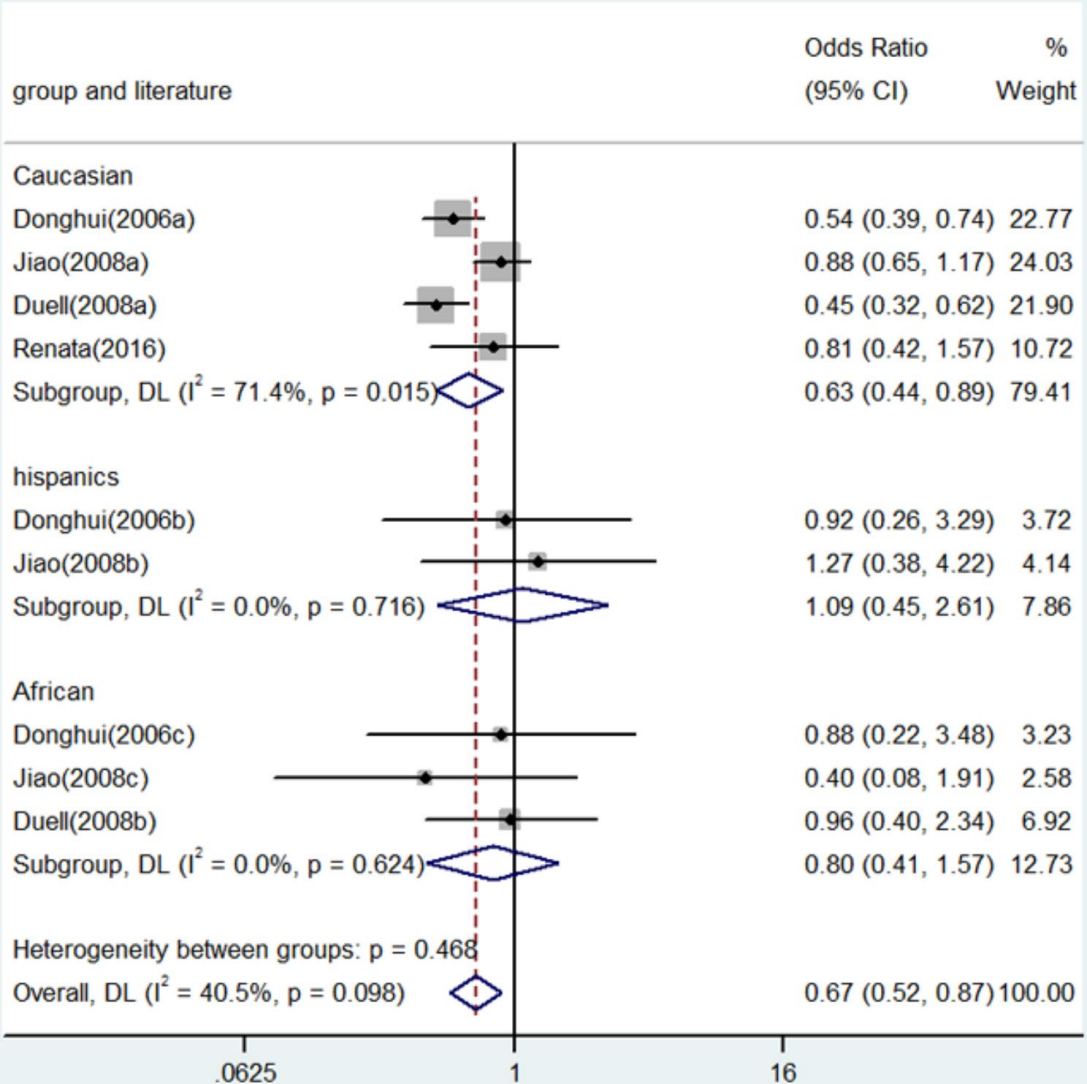
Forest plot for the associations between XRCC3 Thr241Met polymorphism and pancreatic cancer risk through heterozygosis comparison (TC vs. CC). OR, odds ratio; CI, confidence interval

**Fig. 5:**
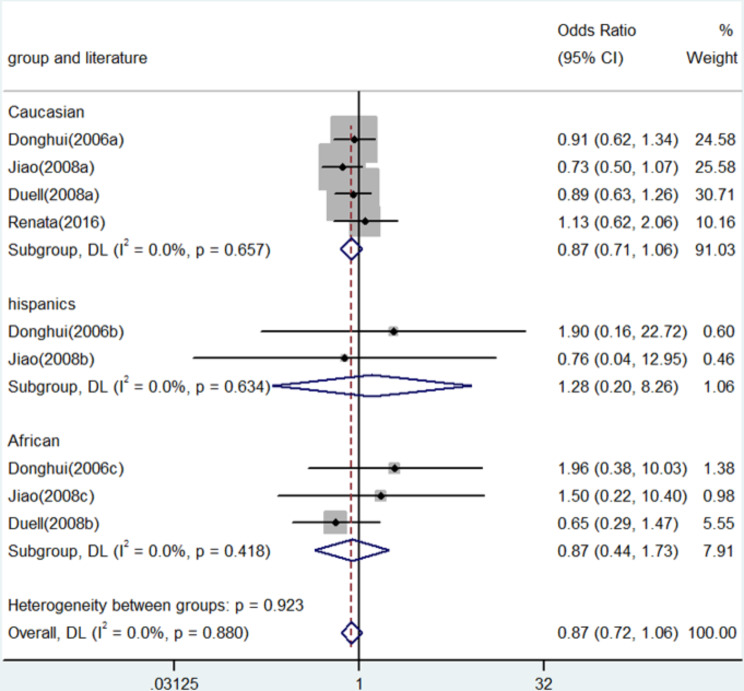
Forest plot for the associations between XRCC3 Thr241Met polymorphism and pancreatic cancer risk through recessive genetic model (TT vs. TC/CC). OR, odds ratio; CI, confidence interval

**Fig. 6:**
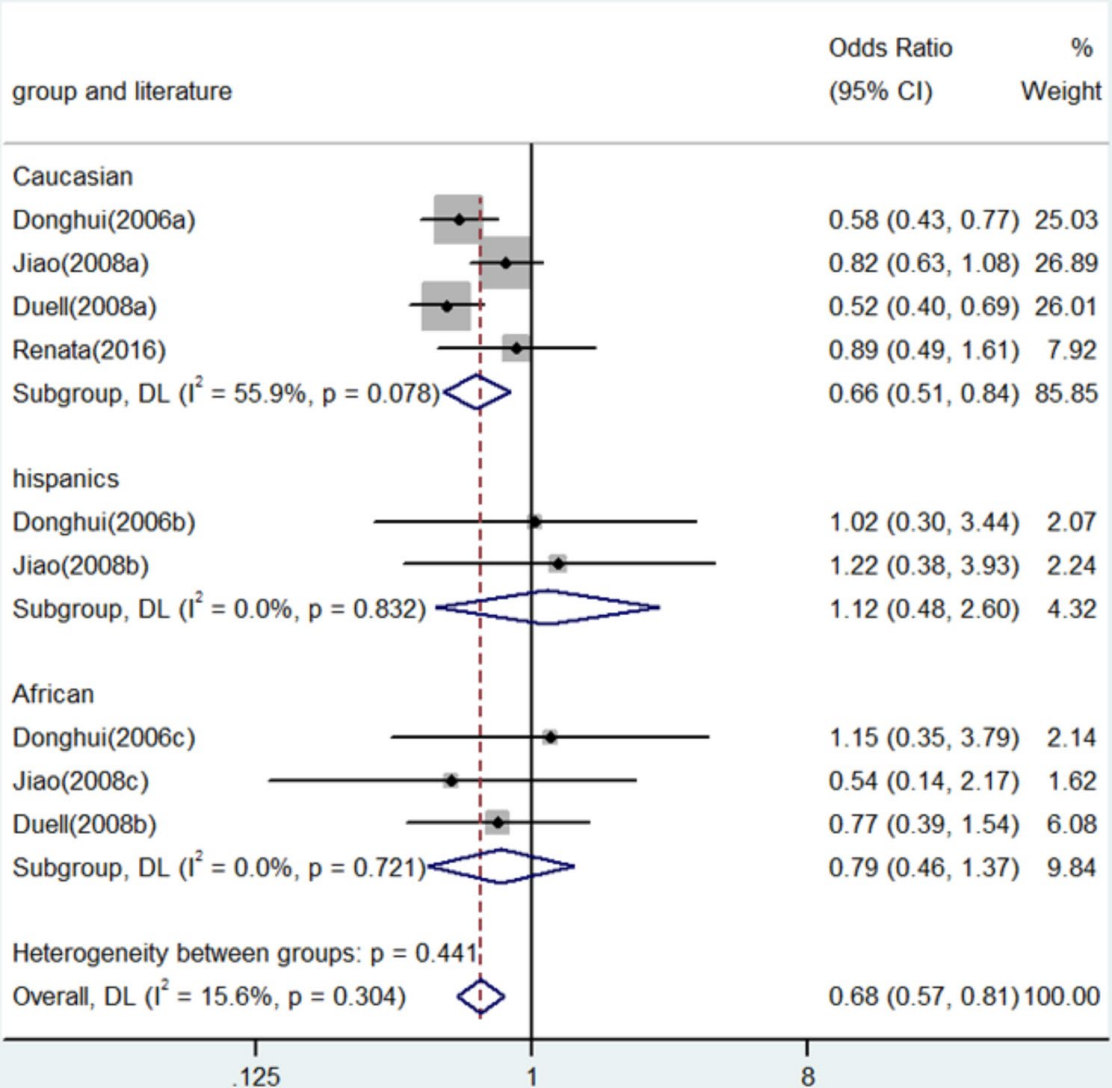
Forest plot for the associations between XRCC3 Thr241Met polymorphism and pancreatic cancer risk through dominate genetic model (TC/TT vs. CC). OR, odds ratio; CI, confidence interval

In numerous genetic studies, a strong correlation was found between the XRCC3 Thr241Met polymorphism and the risk of pancreatic cancer. To be more precise, homozygote comparison (TT vs. CC) demonstrated an OR of 0.71 (95% CI: 0.58–0.88, *P* = 0.001, Fig. 2), whereas allele contrast (T vs. C) revealed an OR of 0.77 (95% CI: 0.70–0.86, *P* < 0.001, Fig. 3). An OR of 0.67 (95% CI: 0.52–0.87, *P* = 0.003) was shown in the heterozygote comparison (TC vs. CC) in Fig. 4. Figure 5 show that the OR in the recessive genetic model (TT vs. CC/TC) was 0.87 (95% CI: 0.72–1.06, *P* = 0.167). Additionally, Fig. 6 showed an OR of 0.68 (95% CI: 0.57–0.81, *P* < 0.001) for the dominant genetic model (TT/TC vs. CC). A full summary of the main findings on the relationship between pancreatic cancer risk and the XRCC3 Thr241Met polymorphism was shown in Table 3

**Table 3:** Meta-analysis of the XRCC3 C18067T polymorphism and pancreatic cancer risk

Comparison	Population	*N*	Test of association			Mode	Test of heterogeneity		
			OR	95%CI	*P*		χ2	*P*	I^2^
T versus. C	Overall	9	0.77	0.70-0.86	0	Fixed	6.73	0.566	0
	Caucasian	4	0.77	0.67-0.88	0	Fixed	4.11	0.250	27.0
	Hispanics	2	1.11	0.57-2.20	0.753	Fixed	0	0.980	0
	African	3	0.83	0.55-1.26	0.506	Fixed	1.31	0.519	0
TT versus. CC	Overall	9	0.71	0.58-0.88	0.001	Fixed	3.14	0.925	0
	Caucasian	4	0.70	0.56-0.87	0.001	Fixed	1.13	0.770	0
	Hispanics	2	1.31	0.19-8.79	0.782	Fixed	0.16	0.693	0
	African	3	0.82	0.40 -1.68	0.590	Fixed	1.27	0.530	0
TC versus. CC	Overall	9	0.67	0.52-0.87	0.003	Random	13.43	0.098	40.5
	Caucasian	4	0.63	0.44-0.89	0.008	Random	10.47	0.015	71.4
	Hispanics	2	1.09	0.45-2.61	0.848	Fixed	0.13	0.716	0
	African	3	0.80	0.41-1.57	0.517	Fixed	0.94	0.624	0
TT versus. TC/CC	Overall	9	0.87	0.72-1.06	0.167	Fixed	3.74	0.923	0
	Caucasian	4	0.87	0.71-1.06	0.173	Fixed	1.61	0.657	0
	Hispanics	2	1.28	0.20-8.26	0.797	Fixed	0.34	0.634	0
	African	3	0.87	0.44-1.73	0.697	Fixed	1.74	0.418	0
TT/TC versus. CC	Overall	9	0.68	0.57-0.81	0	Fixed	9.48	0.441	15.6
	Caucasian	4	0.66	0.51-0.84	0.001	Random	6.80	0.078	55.9
	Hispanics	2	1.12	0.48-2.60	0.793	Fixed	0.04	0.832	0
	African	3	0.79	0.46-1.37	0.409	Random	0.65	0.721	81.7

OR, odds ratio; CI, confidence interval

### Evaluation of between-study heterogeneity

Overall, the analysis revealed minimal heterogeneity across most genetic models. Notable exceptions were observed in the heterozygote comparison (χ² = 10.47, *P* = 0.015, I² = 71.4, Table 3) and the dominant genetic model (χ² = 6.80, *P* = 0.078, I² = 55.9, Table 3), where significant heterogeneity was detected. Meta-regression analysis pinpointed sample size as a critical factor contributing to this heterogeneity. Specifically, the study conducted by Renata et al. introduced substantial heterogeneity, attributable primarily to its relatively small sample size.

### Subgroup analysis and publication bias

Subgroup analysis was meticulously performed based on ethnicity, revealing significant findings particularly within the Caucasian population. Comprehensive details of these findings are available in Table 3. The funnel plot analysis showed no apparent asymmetry, and the reliability of these results was further supported by Egger’s test, which yielded a non-significant bias (*P* = 0.560).

## Discussion

The etiology of pancreatic cancer remains a topic of ongoing research, with current consensus suggesting a multifactorial influence spanning environmental, hereditary, demographic, lifestyle, genetic, and psychosocial factors. Environmental elements, particularly lifestyle choices such as smoking, exposure to secondhand smoke, and excessive alcohol consumption, are notably impactful in the incidence and progression of pancreatic cancer.

Moreover, psychological and demographic factors are acknowledged to modulate the risk of pancreatic cancer. The varying susceptibility within populations, despite similar environmental risk exposures, underscores the significance of genetic predisposition in the disease’s onset and progression. The role of genetic factors, especially at the single nucleotide polymorphism (SNP) level, is increasingly recognized in the pathogenesis of pancreatic cancer. Susceptibility genes of interest primarily include oncogenes, tumor suppressor genes, and DNA repair genes, with their polymorphisms accounting for individual variations in disease response and progression due to environmental factors.

XRCC3, a crucial DNA repair gene, functions as a molecular scaffold, facilitating single-strand break repair and base excision repair by binding to repair-related proteins. Epidemiological data increasingly suggest a link between repair gene polymorphisms and cancer risk, with XRCC3 implicated in various malignancies, including lymphoma, lung, esophageal, salivary gland, colorectal, cervical, breast, and stomach cancers [21–28]. Our findings indicate a decreased risk in Caucasian populations, marking this as potentially the first meta-analysis to focus on this aspect. However, the current results indicate that XRCC3 Thr241Met polymorphism is not associated with the risk of pancreatic cancer in Hispanics population or African population. We think the different results from different population are not surprising, because the results of genetic polymorphism are influenced by many aspects such as geography, ethnicity and environment. And even within the same continent or within the same country, there can still be racial differences. China, Iran, India, Saudi Arabia and other countries are all in Asia, but there are obvious differences in their race. Furthermore, even if different studies study the same race, they may get different results due to the difference in sample size, which is also the reason for our meta-analysis. By conducting Between-Study Heterogeneity and meta-regression, we successfully identified the source of heterogeneity and reduced the impact of heterogeneity to a very low level through subgroup analysis, thus ensuring the reliability of the meta-analysis results. Finally, the sensitivity analysis and publication bias also show that our results are very convincing.

However, several limitations warrant consideration. Primarily, the inclusion of more diverse studies, particularly from Asian, African, and other ethnic groups, would enhance the meta-analysis. Additionally, the influence of various confounders could not be fully assessed due to insufficient data, and the overall participant number in our study is relatively small.

In summary, our findings suggest that the XRCC3 Thr241Met polymorphism is associated with a reduced risk of pancreatic cancer in Caucasian populations. Further research is imperative to corroborate these findings.

### Data sharing statement

All data generated or analyzed during this study are included in this published article.

## Data Availability

No datasets were generated or analysed during the current study.
